# Connecticut

**Published:** 1896-06

**Authors:** 


*Connecticut.* Gratifying progress in controlling tuberculosis in this State under the present laws is reported. Although the use of tuberculin as a diagnostic agent is optional with cattle-owners, the applications for the test come in more rapidly than can be attended, and from many who a year ago were opposed to this plan of determining the existence of the disease. Tuberculin has proved an absolutely reliable test, and no errors are charged to its account as yet. The destruction of the animals has been done in public, unless objected to by the owner, and this plan of education has proved effective and won over many friends to the mode of extermination.

